# Recovery of Chloride Penetration Resistance of Cement-Based Composites Due to Self-Healing of Cracks

**DOI:** 10.3390/ma14102501

**Published:** 2021-05-12

**Authors:** Kyung Suk Yoo, Seung Yup Jang, Kwang-Myong Lee

**Affiliations:** 1 Department of Transportation System Engineering, Graduate School of Transportation, Korea National University of Transportation, 157, Chuldo-bangmulgwan-ro, Uiwang 16106, Korea; yooks815@ut.ac.kr; 2 Department of Civil, Architectural, and Environmental Systems Engineering, Sungkyunkwan University (SKKU) 2066, Seobu-ro, Jangan-gu, Suwon 16419, Korea; leekm79@skku.edu

**Keywords:** cement-based composites, crack, self-healing, chloride, electrical migration–diffusion test, surface coating

## Abstract

This study proposed a method of applying coating on uncracked surfaces of test specimens in the electrical migration–diffusion test for the evaluation of the chloride penetration resistance of cracked cement-based composites. It was shown that, by applying the proposed method, the recovery of the chloride penetration resistance from self-healing of cracks can be evaluated more accurately because the application of surface coating reduces the test time and the error introduced by over-simplification. Based on observations of the self-healing-induced recovery of chloride penetration resistance, a phenomenological model for predicting the progress of crack self-healing in cement-based composites was suggested. This model is expected to evaluate the chloride penetration resistance more accurately in actual concrete structures with cracks.

## 1. Introduction

Concrete is a construction material with very high durability and excellent mechanical properties but is vulnerable to the formation of cracks owing to various physicochemical actions. Concrete cracks degrade structural durability by providing penetration paths for substances such as water and chloride ions [1]. To prevent the penetration of harmful substances through cracks and subsequent degradation, repair is generally performed when cracks larger than an allowed size occur. Crack maintenance, however, requires considerable cost and time [2]. In addition, cracks tend to propagate and additional cracks often occur even after repair, leading to a cycle of continuous degradation of concrete structure durability performance and a shortening of service life [3].

To reduce the need for crack maintenance, a number of studies on self-healing concrete that recovers durability and mechanical performance through the self-healing of cracks have recently been conducted [4]. Concrete is essentially capable of naturally healing cracks; when moisture is supplied through a crack, it will heal through the hydration of unhydrated cement and the carbonation of calcium hydroxide via a process known as natural healing [5]. This process, however, will only heal very fine cracks [6,7]. Previous studies reported improvements in crack self-healing capacity through the use of inorganic materials [8], super absorbent polymers (SAPs) [9], encapsulated polymers [10], and bacteria [11,12]. Inorganic materials improve the self-healing capacities of cement-based materials through the rehydration of unhydrated cement [13], the generation of calcium carbonate via the carbonation reaction of Ca^2+^ [14,15], or the generation of C–S–H via pozzolanic reactions using fly ash or blast furnace slag [16,17,18,19]; cracks can also be healed through the formation of ettringite within cracks using calcium sulfoaluminate (CSA) and crystalline admixtures [20]. SAPs absorb large quantities of water that are subsequently released during the cement hydration process. When a crack occurs, they absorb the water that comes through the crack and expand to physically block the crack. They are known to further improve healing performance by accelerating the hydration of unhydrated cement through the slow discharge of absorbed water [21,22,23]. Bacteria heal cracks through a phenomenon in which CO_2_ generated by their metabolic activity forms CaCO_3_ crystals through reaction with Ca(OH)_2_ in hardened cement paste [11,12]. Bacteria spores and nutrients dissolve into water that are infiltrated through cracks and the spores subsequently initiate metabolic activity [24,25].

The use of self-healing concrete improves the durability performance and lengthens the target service life of concrete without requiring costly and time-consuming crack repair. To enable commercialization of self-healing concrete, however, a method for quantitatively evaluating the self-healing capacities of different technologies is necessary. In particular, because cracks have a direct impact on durability performance rather than on mechanical properties, it is very important to evaluate the recovery of durability performance through self-healing. In previous studies, crack healing was evaluated using nondestructive testing (NDT) or microstructure analysis [26]. NDT, which has been carried out using radiation testing [23], acoustic emission [27], ultrasonic testing [28,29], and image analysis [30], is a relatively simple process but has a low degree of reliability and limited applicability in directly evaluating mechanical or durability performance. Alternatively, water permeability tests have been applied in the direct evaluation of recovery of durability performance through self-healing [4,6]. To evaluate the reduction in permeability induced by self-healing, Van Mullem et al. [31] introduced water from a water tank in which the water level was held constant to the inside of a cracked specimen and measured the amount of water discharged through the cracks. Gwon et al. [32] evaluated the permeability of a cylindrical specimen with artificially induced cracks installed within a water permeability test cell in terms of the total amount of water that permeated the cracks over a specific period of time. Instead of water permeability testing, water absorption test has also been investigated [33,34]. Recently, pre-standard testing methods for water permeability and absorption have been evaluated by means of an interlaboratory testing campaign [35]. However, water permeability or absorption testing methods have limited applicability in the direct evaluation of resistance to chloride penetration. Therefore, methods for directly evaluating resistance to chloride penetration have been studied [10,36,37,38,39,40]. Van Belleghem et al. [36] confirmed the formation of layers that block against direct chloride penetration through cracks in self-healing concrete in which encapsulated polyurethane is used as a healing material and evaluated self-healing capacity using accelerated chloride diffusion testing. However, the accelerated chloride diffusion test they applied is slow, making it difficult to evaluate self-healing capacity at a specific time. Şahmaran et al. [37] investigated resistance to chloride penetration in specimens with crack widths ranging from 50 to 140 μm using the test method defined under ASTM C 1202 [41] and evaluated the self-healing capacities of the specimens after 60 days of healing age. The ASTM C 1202 method indirectly evaluates resistance to chloride penetration by measuring the quantity of charge passing over a period of 6 h using an applied electrical potential. Despite its short testing time, this method cannot be used to directly measure ion penetration rates because it evaluates electrical conductivity in terms of quantities of charge. This method also has difficulties in effectively evaluating chloride penetration resistance, particularly in self-healing concretes containing inorganic materials, because the electrical conductivity depends on the types and concentrations of ions within the concrete. To overcome these problems, Abro et al. [40] applied a steady-state chloride ion electrical migration–diffusion test for calculating the rate of chloride ion penetration through cracks and used it to evaluate the self-healing capacity of inorganic material-based self-healing cement mortar. They reported that the application of an electrical potential of 36 V can reduce the testing time for a mortar specimen with a water-binder (w/b) ratio of 0.40 by up to 36 h. However, it is encouraged to further reduce the testing time for minimizing the error. In addition, as the cracks in the specimens used in the migration–diffusion testing differ from actual cracks in structures in terms of internal crack geometry, the migration-diffusion test applied for the specimens has some limitations in evaluating the recovery of chloride penetration resistance through self-healing in actual cracks. Hence, it is required to develop a model for evaluating chloride penetration resistance in actual concrete structures.

This study proposes an improved method applying coating on uncracked surfaces of test specimens in the using electrical migration–diffusion test for the more rapid and accurate evaluation of the chloride penetration resistance of cracked cement-based composites. Based on observations of the self-healing-induced recovery of chloride penetration resistance following application of the proposed method, a phenomenological model for evaluating chloride penetration resistance in actual concrete structures is proposed.

## 2. Steady-State Migration—Diffusion Test

As noted in the preceding section, the electrical migration–diffusion tests have been used to rapidly evaluate chloride penetration rates within a short period of time. Standardized electrical migration–diffusion test methods include ASTM C 1202 [41], NT Build 355 [42], and NT Build 492 [43]. The ASTM C 1202 test method indirectly evaluates chloride penetration resistance by measuring the quantity of charge passed charges for 6 h. As previously noted, however, despite the benefit of short testing time, this method cannot directly measure durability parameters such as the chloride ion diffusion coefficient. By contrast, the test specified under NT Build 355 can determine diffusion coefficients using steady-state electrical migration–diffusion testing and the test specified under NT Build 492 can determine non-steady-state chloride ion diffusion coefficients using a non-steady-state test. The steady-state diffusion coefficient can be calculated using the Nernst–Planck equation by measuring the rate of change in chloride ion concentration within a solution inside a diffusion cell after reaching the steady state. On the contrary, the non-steady-state diffusion coefficient measurement test is terminated before reaching the steady state. Instead of measuring the change in chloride ion concentration, the non-steady-state diffusion coefficient is obtained by cutting the specimen perpendicular to the chloride ion penetration direction, spraying the cut with AgNO_3_ solution, measuring the chloride ion penetration depth by observing the color change [43], and applying the equation proposed by Tang and Nilsson [44].

**Figure 1:**
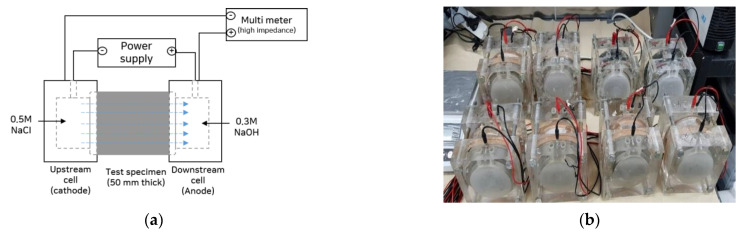
(**a**,**b**) Test set-up for chloride ion migration–diffusion test (ASTM C 1202).

However, it is difficult to use the non-steady-state diffusion coefficient test to calculate only the diffusion coefficient of cracked concrete because the chloride ion penetration depths at crack and in uncracked section differ. For this reason, many researchers have evaluated the steady-state diffusion coefficients of cracked specimens by applying the test setups described under ASTM C 1202 or NT Build 355 [1,38,39,40]. Abro et al. [40] conducted steady-state chloride ion migration–diffusion testing using the ASTM C 1202 set-up shown in Figure 1 and proposed a method for evaluating self-healing capacity by calculating the quantity of chloride ions that moved through the cracks.

If the movement of chloride ions by diffusion is neglected in the electrical migration–diffusion test, the chloride ion flux can be expressed using the following Nernst–Planck equation:

(1) $$J_{c}=D\frac{zF}{RT}c\frac{\partial U}{\partial x},$$

where $J_{c}$ is the chloride ion flux, z is the ionic valence, F is the Faraday constant (=96,485 C per equivalent), R is the gas constant (=8.3145 J/mol·K), T is the absolute temperature (K), D is the diffusion coefficient, c is the chloride ion concentration, U is the electrical potential applied (V), and x is the distance.

The “steady-state condition” refers to the state, occurring after a certain period of time during which ions move by an electrical potential between two cells, in which the chloride ion concentration within the specimen is saturated and, therefore, the incoming and outcoming ion flows per unit time become equal:

(2) $$\frac{\Delta c_{1}}{\Delta t}\approx \frac{\Delta c_{2}}{\Delta t}.$$

In this instance, if the electrical potential is constant, the steady-state diffusion coefficient can be calculated using the Nernst–Planck equation as follows [44]:

(3) $$D_{ssm}=\frac{RTL}{zFU}\frac{V}{A}\frac{1}{c_{1}}\left|\frac{\Delta c_{1}}{\Delta t}\right|=\frac{RTL}{zFU}\frac{V}{A}\frac{1}{c_{1}}\left|\frac{\Delta c_{2}}{\Delta t}\right|,$$

where $c_{1}$ is the concentration in the upstream cell, $c_{2}$ is the concentration in the downstream cell, L is the specimen thickness, V is the cell volume, and A is the cross-sectional area of the specimen. The chloride ion concentration in the upstream cell ($c_{1}$) continues to decrease over the course of an actual test. Therefore, Equation (3) can be converted to the following form in which the diffusion coefficient is defined in terms of the rate of change of the logarithmic value of the chloride ion concentration:

(4) $$D_{ssm}=\frac{RTL}{zFU}\frac{V}{A}\left|\frac{\Delta \ln\left(c_{1}\right)}{\Delta t}\right|.$$

In the formulation in Equation (4), the state in which the rate of change in the logarithmic chloride concentration becomes constant is defined as the quasi-steady-state condition [1,45].

**Figure 2:**
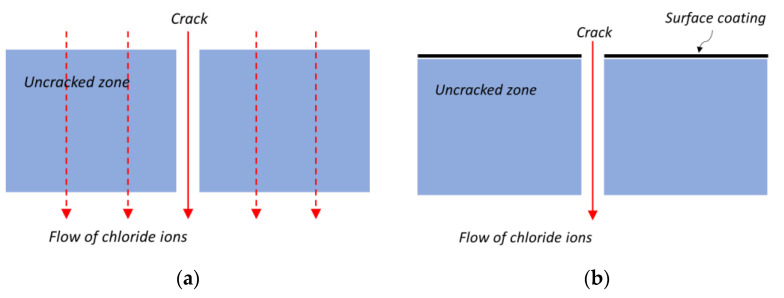
Transport paths of chloride ions in uncoated and coated specimens: (**a**) uncoated specimen and (**b**) coated specimen.

In several previous studies [1,40,46], the penetration paths of chloride ions were divided into crack and uncracked zone as shown in Figure 2a. Under the formulation using a parallel model, the total amount of chloride ion penetrations becomes the sum of the penetrations through the crack and uncracked zone:

(5) $$A_{tot}J_{tot}=A_{ucr}J_{ucr}+A_{cr}J_{cr}$$

here, $A_{tot}$ is the total area of the specimen; $A_{ucr}$ is the area of the uncracked zone of the specimen; $A_{cr}$ is the area of cracking; and $J_{tot}$, $J_{ucr}$, and $J_{cr}$ are the total flux and the fluxes in the uncracked zone and the crack, respectively. If the electrical potential ∂U/∂x acting on the crack and uncracked zone is assumed to be same and constant, the following relationship holds under the Nernst–Planck equation [46]:

(6) $$A_{tot}D_{meas}=A_{cr}D_{cr}+A_{ucr}D_{ucr},$$

where $D_{meas}$ is the measured chloride ion diffusion coefficient of the specimen, $D_{ucr}$ is the chloride ion diffusion coefficient in the uncracked zone, and $D_{cr}$ is the diffusion coefficient in the crack. Assuming $A_{ucr}\approx A_{tot}$, the chloride ion diffusion coefficient in the crack is given as:

(7) $$D_{cr}=\frac{A_{tot}}{A_{cr}}\left(D_{meas}-D_{ucr}\right).$$

To determine the chloride ion penetration rate in the crack using Equation (7), it is necessary to obtain the diffusion coefficient in the uncracked zone. In previous studies [1,40], the diffusion coefficient in the uncracked zone was assumed based on chloride ion diffusion coefficients measured from other uncracked specimens. However, the assumption that the diffusion coefficient of a specimen without cracking will be the same as that of the uncracked zone of a cracked specimen is questionable; this is discussed in more detail in our analysis of the results in Section 4. To re-assess the validity of the parallel model hypothesis and calculate the chloride ion diffusion coefficient in the crack more accurately, we decided in this study to apply epoxy coating to the external contact surfaces in the uncracked zone, as shown in Figure 2b. If the amount of the chloride ions penetrating a specimen through the internal surfaces in the crack via diffusion can be assumed to be negligibly small compared to the amounts of the chloride ions that penetrate through crack via migration. This makes it possible to assume that chloride ions penetrate only through crack. If this assumption is valid, the chloride ion diffusion coefficient of the crack can be directly obtained from the measured diffusion coefficient of the specimen as follows:

(8) $$D_{cr}=\frac{A_{tot}}{A_{cr}}D_{meas}.$$

## 3. Experimental Program

### 3.1. Materials and Mixture Proportions

Mortar specimens with water-to-binder ratios (w/b) of 0.4 were prepared for testing. In addition to an ordinary Portland cement (OPC) mixture, two self-healing mixtures containing ground granulated blast furnace slag (GGBFS) and clinker with a particle size larger than that of OPC were formulated. In both mixtures, 25% of the OPC was replaced with GGBFS. Clinker with a particle size of 0.85 mm or below replaced 5% (or 10%) of the OPC and clinker with a particle size of 2.5 mm or below replaced 5% (or 10%) of the fine aggregate. Na_2_SO_4_ and anhydrate gypsum were added as a GGBFS stimulant to each mixture at 1.5% of the binder weight. Clinker with a particle size larger than that of the cement is expected to generate self-healing because the insides of the clinker particles are in the unhydrated state and generate hydrates by reacting with water that penetrates through formed cracks [47]. Unreacted GGBFS also contributes to self-healing by generating C–S–H through reactions with water and Ca(OH)_2_. Table 1 lists the mixture proportions in detail.

### 3.2. Preparation of Test Specimens

After mixing the materials according to the mixture proportions listed in Table 1, the mixtures were poured into cylindrical molds with a diameter of 100 mm and a height of 200 mm and compacted using a vibrator to prepare specimens. Following air curing for 24 h, the specimens were removed from the mold and subjected to water curing for 28 days.

**Figure 3:**
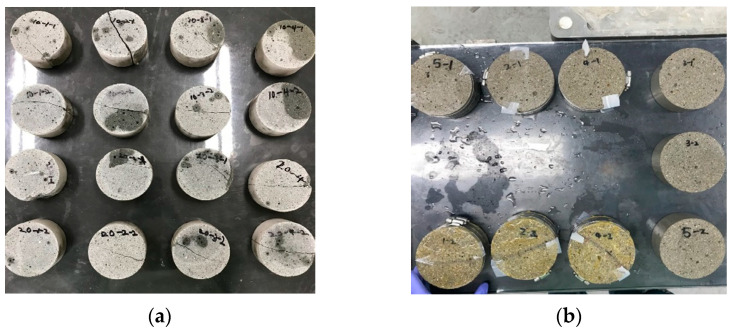
Uncoated and surface-coated specimens: (**a**) uncoated specimens and (**b**) surface-coated specimens.

After 28 days, each specimen was cut with both ends excluded to form two disk specimens with lengths of 50 mm each. The disk specimens were split using a compression testing machine and the surface coating was then applied. After that, silicone tapes with thicknesses corresponding to the target crack widths were attached to both ends of each crack. The split specimens were then fixed using a steel clamp, as shown in Figure 3. The details of our method for preparing cracked specimens can be found in [40]. The actual crack widths were measured twice at three points with 30 mm spacing along crack on the top and bottom faces of each specimen using an optical microscope immediately after re-assembling of split specimens. The mean value was taken as the crack width of the specimen. Table 2 lists the measured crack widths of the prepared cracked specimens. It is seen from the table that the prepared cracks had errors in width of up to 10% of the target crack widths. For each mixture, a total of seven specimens were fabricated, including one uncracked specimen without coating, and three uncoated and three coated specimens with induced crack widths of 0.1, 0.2, and 0.3 mm, respectively. Because only one specimen was tested for each crack width, coating condition, and mixture type, it has some limitations in capturing the statistical uncertainties included in the test results. However, the main focus of this study is to assess the differences that come from the application of surface coating, and it can be done by directly comparing the results of uncoated and coated specimens, which were manufactured from the same cylinder. The specimen coatings were obtained by applying epoxy resin onto all surfaces facing upstream cell except for crack (refer to Figure 3b). The repeatability of the test has been checked in the previous study [40]. In this study, the test was done on one specimen for each crack width and mixture.

### 3.3. Test Methods

The upstream and downstream cells were filled with 0.5 M NaCl and 0.3% NaOH solutions, respectively, as shown in Figure 1 for the chloride migration-diffusion test. To induce the movement of chloride ions, a voltage of 36 V was applied. The rates of change in the chloride ion concentrations in the upstream and downstream cells were obtained by measuring the concentrations at regular intervals using an ion-selective electrode. The concentrations were measured every 20 min for 2 h after the start of the test, every 60 min for the next 4 h, and every 120 min thereafter until the end of the test. When each diffusion coefficient measurement test was completed, the specimen was subjected to water curing until the next measurement. The test was conducted four times, at 0, 28, 56, and 91 days of healing age.

## 4. Results and Discussion

### 4.1. Variation of Chloride Ion Concentration and Time to Reach the Quasi-Steady State

**Figure 4:**
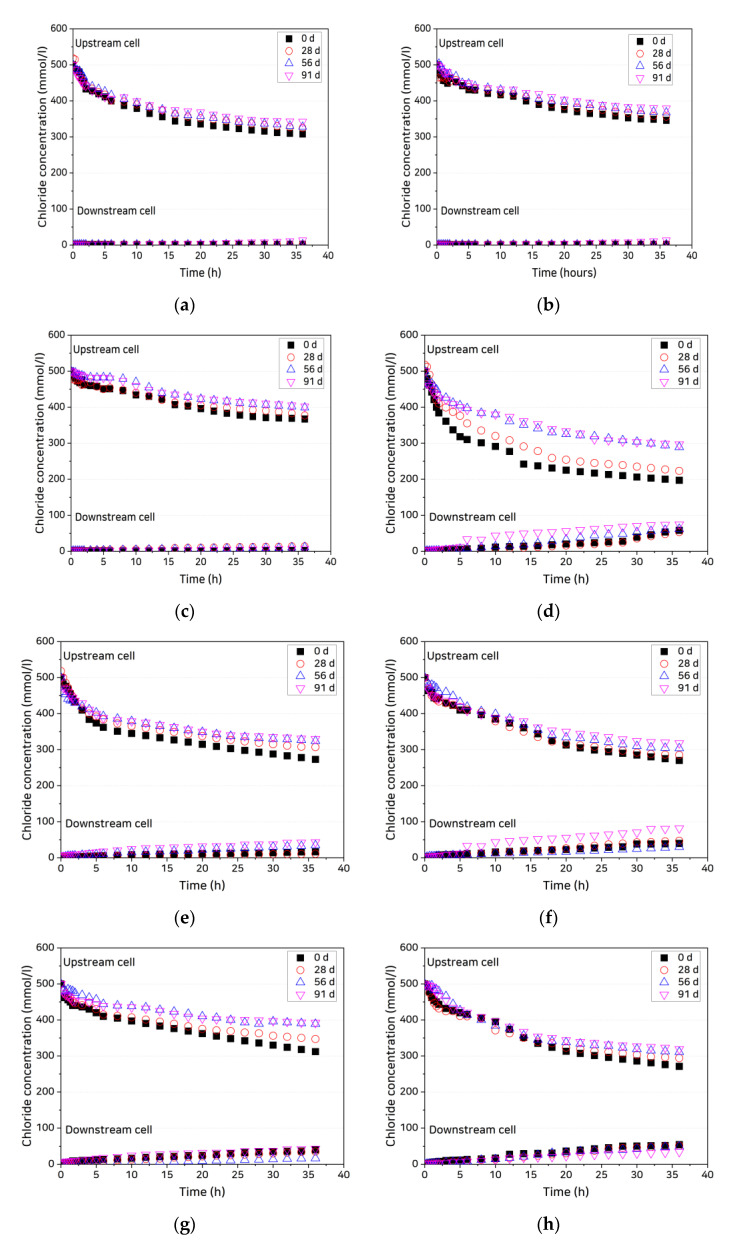

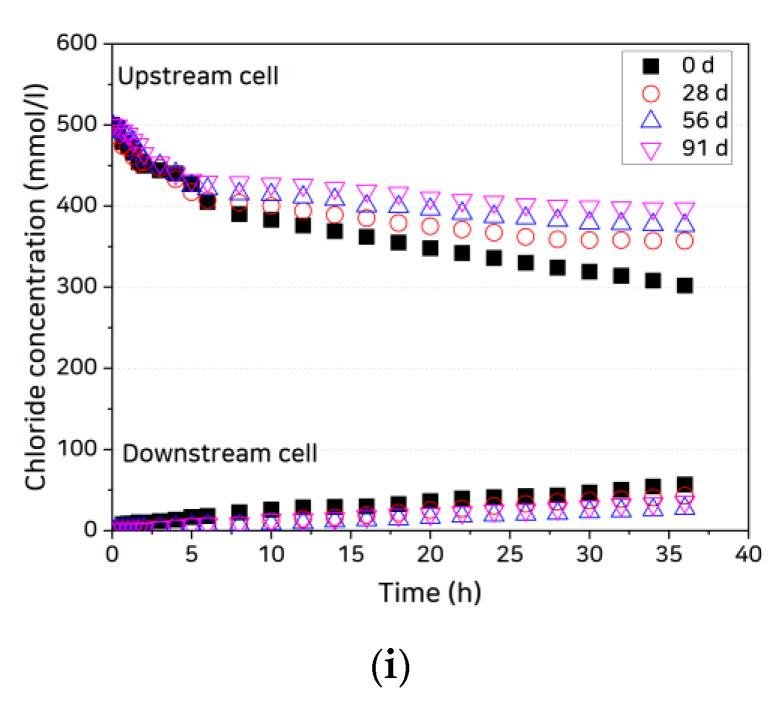
Variation in chloride concentrations in upstream and downstream cells: (**a**) OPC, uncracked; (**b**) SHC15, uncracked; (**c**) SHC30, uncracked; (**d**) OPC, crack width 0.3 mm, uncoated; (**e**) OPC, crack width 0.3 mm, coated; (**f**) SHC15, crack width 0.3 mm, uncoated; (**g**) SHC15, crack width 0.3 mm, coated; (**h**) SHC30, crack width 0.3 mm, uncoated; and (**i**) SHC30, crack width 0.3 mm, coated.

Figure 4 shows the changes over time of the upstream- and downstream-cell chloride ion concentrations in the uncracked and cracked (0.3-mm crack width) OPC and SHC30 specimens. In the upstream cell, the chloride ion concentrations rapidly decrease at first and then undergo a slower decrease with an almost constant slope. The cracked specimens exhibit higher concentrations than the uncracked specimens. In the downstream cell, the chloride ion concentration increases as the chloride ions passing through the crack and uncracked zone reach the cell, after which the slope also becomes constant. In the uncracked specimens, the concentration in the downstream cell slowly increases as the time at which the slope of concentration change becomes constant is approached. In the cracked specimens, by contrast, the passage of chloride ions through the cracks causes the ion concentrations to increase immediately after the start of testing, and the changes in slope are induced by chloride ions passing through the uncracked zone of the specimens. In addition, although the slopes of concentration change in the upstream and downstream cells converge, they do not become identical. Because the slopes vary slightly even after a considerable time, it is not easy to clearly determine when the steady state is reached.

**Figure 5:**
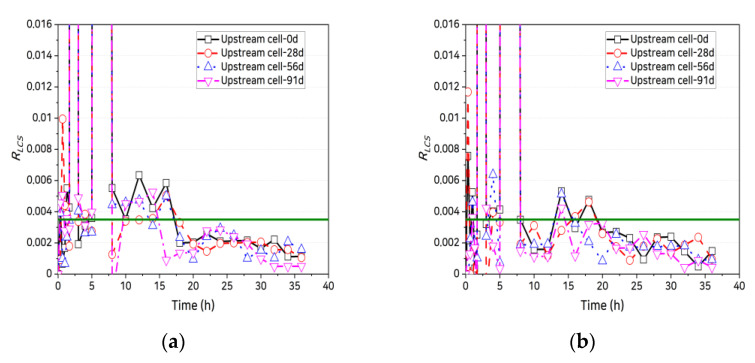

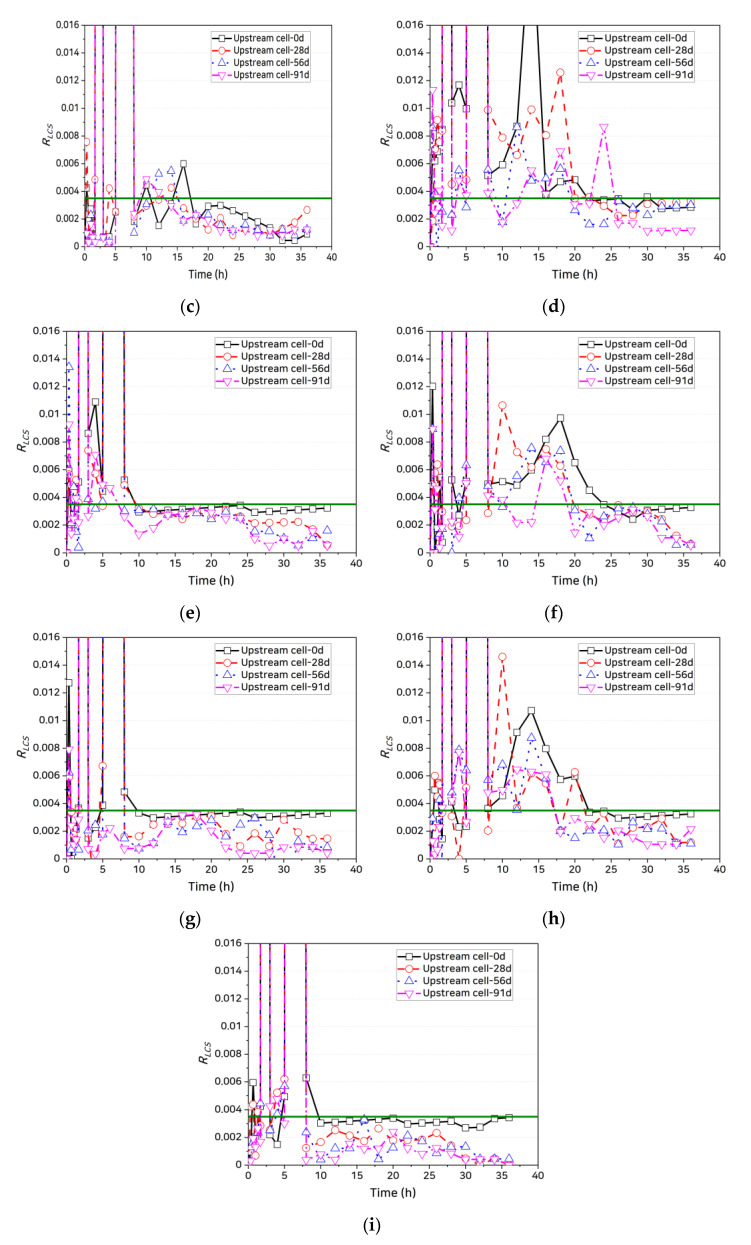
Variation in rate of change of chloride concentration: (**a**) OPC, uncracked; (**b**) SHC15, uncracked; (**c**) SHC30, uncracked; (**d**) OPC, crack width 0.3 mm, uncoated; (**e**) OPC, crack width 0.3 mm, uncoated; (**f**) SHC15, crack width 0.3 mm, uncoated; (**g**) SHC15, crack width 0.3 mm, coated; (**h**) SHC30, crack width 0.3 mm, uncoated; and (**i**) SHC30, crack width 0.3 mm, coated.

**Figure 6:**
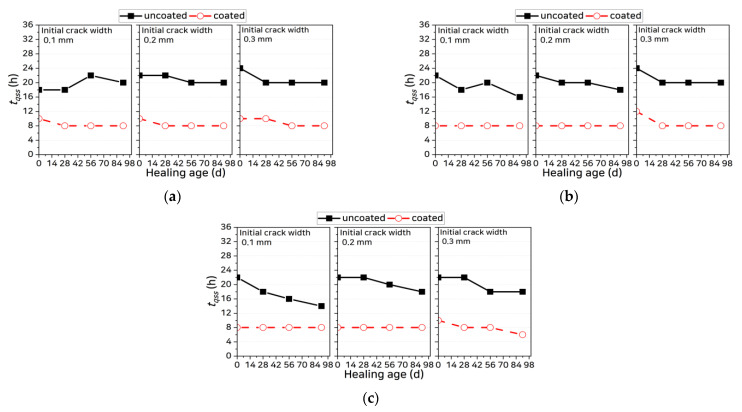
Comparison of times needed to reach the quasi-steady state: (**a**) OPC, (**b**) SHC15, and (**c**) SHC30.

Abro et al. [40] reported that, after reaching the quasi-steady state, the magnitude of the standard deviation of $\Delta c_{1}/c_{1}$ becomes less than 1%. However, determining this is cumbersome because the quantitative criterion for the allowable magnitude of the standard deviation is ambiguous, and the deviation must be examined as the size of the measurement section continuously varies. Accordingly, in this study, the rate of change of the log concentration gradient in the upstream cell was measured to better identify the time needed to reach the quasi-steady state. It was assumed that the quasi-steady state was reached when the rate of change of $\Delta ln(c_{1})/\Delta t$ at each time step became lower than the allowable error, that is:

(9) $$R_{LCS}=\frac{{\left|\frac{\Delta ln\left(c_{1}\right)}{\Delta t}\right|}_{n+1}-{\left|\frac{\Delta ln\left(c_{1}\right)}{\Delta t}\right|}_{n}}{{\left|\frac{\Delta ln\left(c_{1}\right)}{\Delta t}\right|}_{n}}\leq \varepsilon ,$$

where $R_{LCS}$ is the rate of change of the log concentration gradient and the allowable error ε is set to 3.5 × 10^−3^ by referring to the data in Figure 5. The coated specimens reached the quasi-steady state more rapidly than the uncoated specimens. Figure 6 shows the time needed by each specimen to reach the quasi-steady state ($t_{qss}$). For the uncoated specimens, $t_{qss}$ ranged from 14 to 24 h and for the coated specimens, it ranged from 6 to 12 h, a decrease of 44–67% relative to the uncoated specimens. There were, however, no significant differences in $t_{qss}$ in terms of mixing characteristics, age, or crack width.

As noted previously, as crack healing can occur within a short period of time in a self-healing cement composite, it is very important to reduce the test time in evaluating self-healing capacity; correspondingly, surface coating is applied to obtain more accurate self-healing capacity evaluation because it decreases the time needed to reach the quasi-steady state and, therefore, reduces the test time.

### 4.2. Relationship Between Chloride Ion Diffusion Coefficient and Crack Width

**Figure 7:**
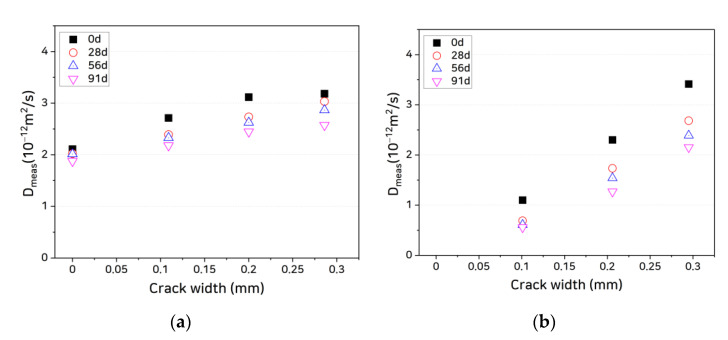

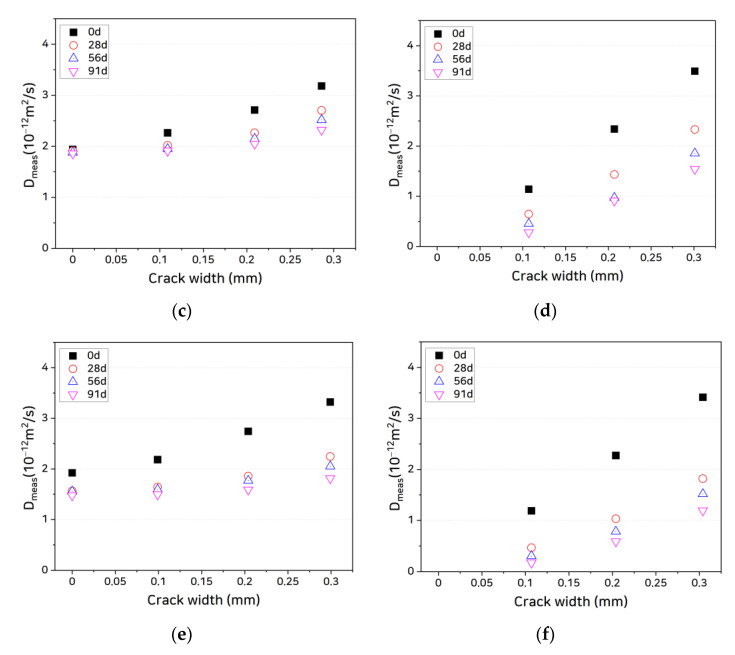
Measured diffusion coefficients of specimens as functions of crack width and healing age: (**a**) OPC, uncoated; (**b**) OPC, coated; (**c**) SHC15, uncoated; (**d**) SHC15, coated; (**e**) SHC30, uncoated; and (**f**) SHC30, coated.

Once the chloride ion log concentration gradient is obtained after reaching the quasi-steady state, the chloride ion diffusion coefficient $D_{meas}$ of a specimen can be calculated using Equation (4). Figure 7 shows the relationship between the crack width and diffusion coefficient as a function of healing age. It is seen that the chloride ion diffusion coefficient of each specimen increases linearly with the crack width, whereas the diffusion coefficient decreases as the age increases. These results confirm those of Abro et al. [40]. It is also noted that a slope of diffusion coefficient to crack width decreases because the reduction in diffusion coefficients is larger as the crack width increases.

**Figure 8:**
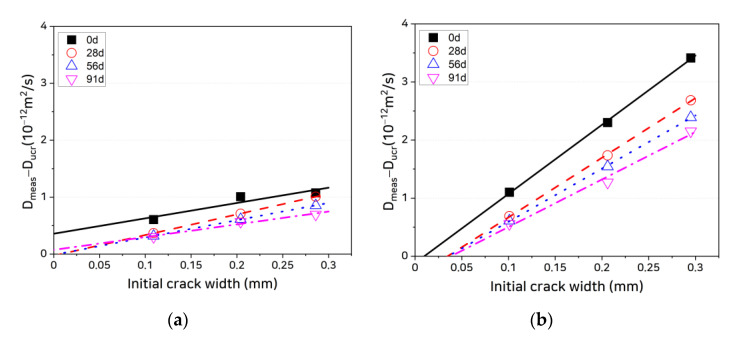

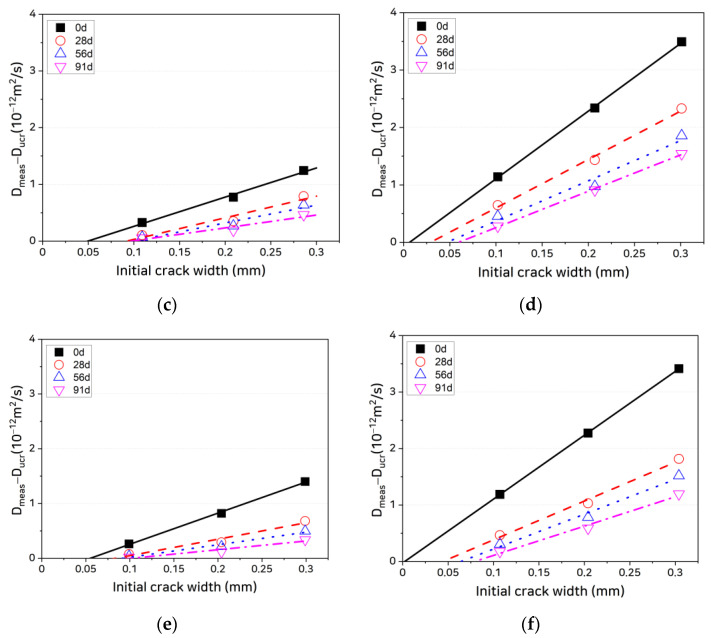
Diffusion coefficient through cracks as a function of crack width: (**a**) OPC, uncoated; (**b**) OPC, coated; (**c**) SHC15, uncoated; (**d**) SHC15, coated; (**e**) SHC30, uncoated; and (**f**) SHC30, coated.

Most noteworthy are the differences in slope of diffusion coefficient to crack width among the specimens. Because ions moved only through the cracks in the coated cracked specimens, the diffusion coefficients calculated using the total areas of these specimens were smaller. Therefore, to compare the differences in the quantities of chloride ions passing through the cracks, we can compare the values obtained by subtracting the diffusion coefficients of the uncracked zone from the diffusion coefficients of the overall specimens, as shown in Figure 8. It is seen from the figure that the slopes of the curves for the uncoated specimens are smaller than those of the coated specimens.

If Equations (6) and (7) under the parallel model described in Section 2 hold, Equation (7) can re-expressed as follows:

(10) $$D_{meas}-D_{ucr}=\frac{A_{cr}}{A_{tot}}D_{cr}=\frac{w_{cr}\;d}{A_{tot}}\beta_{cr}D_{0},$$

where $w_{cr}$ is the crack width, d is the specimen diameter, $\beta_{cr}$ is the crack formation factor representing tortuosity according to the internal crack geometry, and $D_{0}$ is the ion diffusion coefficient in the pore solution, which has a value of 2.03 × 10^−9^ m^2^/s at 23 °C [48]. In this equation, the slope of the relationship between crack width and diffusion coefficient varies depending on $\beta_{cr}$. Although each cracked specimen has, as expected, different internal crack geometries, it is difficult to ascertain whether the differences in slope owing to the application of coating arise from differences in $\beta_{cr}$ given that the slopes of the uncoated specimens are consistently smaller than those of the coated specimens by a factor of close to two. In other words, there is no reason for the slope to change significantly as a result of coating if the assumption applied under the parallel model holds. Consequently, the finding that the uncoated specimens exhibit smaller slopes indicates that the diffusion coefficients of the uncracked zone vary by crack width, which in turn suggests that the assumption applied under the parallel model, i.e., that the electrical potentials ∂U/∂x acting on crack and uncracked zone are the same, is not valid.

According to Gauss’ law, the electrical potential is inversely proportional to the permittivity and directly proportional to the charge density, which, in turn, is proportional to the ion concentration [49]:

(11) $$\nabla^{2}U=\frac{F}{\varepsilon_{0}\varepsilon_{r}}\underset{i}{\sum }c_{i}z_{i},$$

where $\varepsilon_{0}$ ~ 8.8542 × 10^−12^ F/m is the permittivity in vacuum, $\varepsilon_{r}$ is the relative permittivity, and $c_{i}$ and $z_{i}$ are the concentration and valence of ion *i*, respectively. As a saturated crack is filled with water, its permittivity will be much higher than that of cement-based composites. The relative permittivity of water is 81, whereas that of concrete varies depending on the internal porosity and is known to range from approximately 5 to 10 [50]. As a result of this disparity in permittivity, the electrical potential of crack should be much smaller than that of uncracked zone. Under this assumption, Equation (6) must be revised as follows:

(12) $$A_{tot}D_{meas}{\left(\frac{\partial U}{\partial x}\right)}_{tot}=A_{cr}D_{cr}{\left(\frac{\partial U}{\partial x}\right)}_{cr}+A_{ucr}D_{ucr}{\left(\frac{\partial U}{\partial x}\right)}_{ucr}.$$

Under the further assumptions that $A_{ucr}\approx A_{tot}$ and $\partial U_{ucr}/\partial x\approx \partial U_{tot}/\partial x$, Equation (12) can be re-expressed as:

(13) $$D_{meas}-D_{ucr}=D_{cr}\frac{A_{cr}{\left(\frac{\partial U}{\partial x}\right)}_{cr}}{A_{tot}{\left(\frac{\partial U}{\partial x}\right)}_{tot}}.$$

Under Equation (13), the diffusion coefficient through a crack ($D_{meas}-D_{ucr}$) decreases as the crack width increases if the electrical potential of the crack is smaller than that of uncracked zone. In other words, the diffusion coefficient of the uncracked zone increases with the crack width. As Equation (13) is based on a simple assumption, it is difficult to use it to accurately identify changes in the electrical potentials of crack and uncracked zone; for more accurate analysis, it is necessary to examine changes in these electrical potentials using numerical analysis, as in the study conducted by Yang [49]. Nevertheless, it is obvious that the diffusion coefficients through cracks are underestimated in the electrical migration–diffusion testing of uncoated cracked specimens if the crack and non-crack electrical potentials are assumed to be the same. This indicates that more accurate evaluation can be performed by applying coating to the uncracked surface.

### 4.3. Evaluation of Crack Healing Capacity

**Figure 9:**
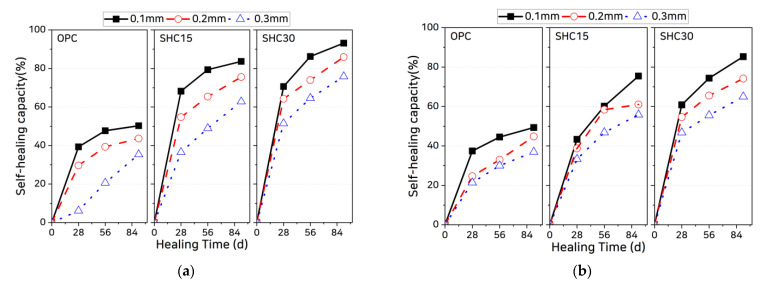
Evaluated self-healing capacities of uncoated and coated specimens: (**a**) uncoated specimens and (**b**) coated specimens.

It is seen from Figure 8 that the reductions in diffusion coefficient are much larger in the mixtures containing self-healing materials (SHC15 and SHC30) than in the OPC and that the reduction in diffusion coefficient increases as the self-healing material content increases. The largest reductions in diffusion coefficient are observed at a healing age of 28 days, after which the rate of reduction slowly decreases. To quantitatively evaluate the chloride penetration resistance arising from self-healing, the self-healing capacity can be calculated, following the method proposed by Abro et al. [40], as a function of the diffusion of chloride ions through the crack:

(14) $$SH=1-\frac{D_{meas}\left(t\right)-D_{ucr}\left(t\right)}{D_{meas}\left(0\right)-D_{ucr}\left(0\right)},$$

where t is the healing age. For the coated specimens, $D_{ucr}$ = 0. Figure 9 shows the self-healing capacities calculated using Equation (14). For both the uncoated and coated specimens, the self-healing capacities tend to increase with the content of self-healing material and tend to decrease as the initial crack width increases. These results are in close agreement with those of Abro et al. [40], although it is important to note that the self-healing capacities of the uncoated specimens are greater than those of the coated specimens. This appears to be because the diffusion coefficient of the uncracked zone of the cracked specimen was found to be higher than that of the uncoated specimens and, therefore, the values of $\left(D_{meas}-D_{ucr}\right)$ were lower, as described previously. Because the initial values at the beginning of healing (day 0) were found to be small, the self-healing capacities relative to the initial values were larger. This suggests that the self-healing capacities of the uncoated specimens are overestimated, whereas those of the coated specimens are accurately estimated because it is possible to directly calculate the chloride ion diffusion coefficient through crack.

### 4.4. Phenomenological Model for Crack Healing Process and Recovery of Resistance to Chloride Ion Penetration

#### 4.4.1. Concept of Healed Crack Width

Previous studies have found that, if the crack width is below a certain value (the “critical crack width” [1,46]), the ion diffusion rate in the crack will be the same as that for concrete without cracking. The size of the reported critical crack width depends somewhat on the study but has been found to lie within the 55–100 μm range [1,37,46,51,52]. However, the cause of the critical crack width phenomenon has yet to be clearly identified.

In the test results obtained in this study, the *x*-axis intercepts of the straight lines connecting the $D_{meas}-D_{ucr}$ values at each crack width in Figure 8 represent the critical crack widths. For the coated specimens, the critical crack widths at day 0 of healing age are very small (less than 10 μm) but increase with the healing age. This supports the following hypotheses:

- The critical crack width is the result of self-healing.
- The critical crack width is not fixed but varies depending on the healing age with a rate of change that depends on the self-healing capacity.

**Figure 10:**
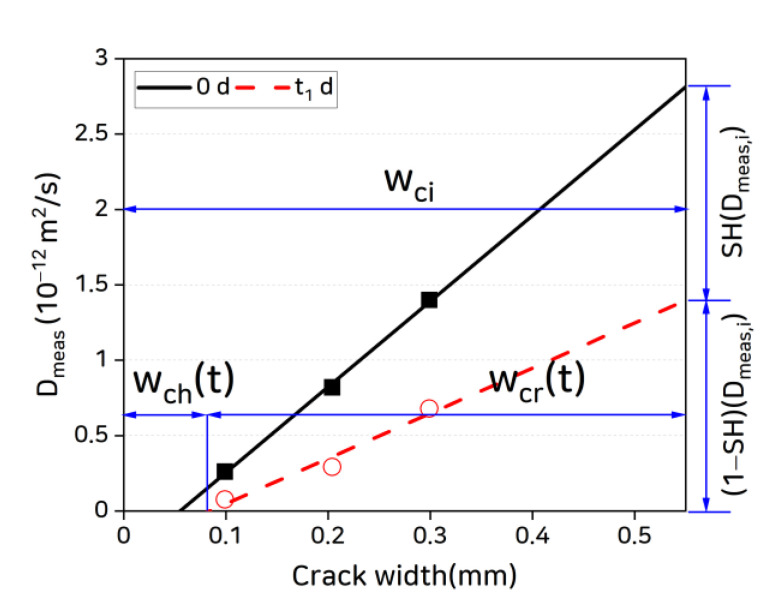
Healed crack width concept.

Critical crack widths that were found in previous studies appeared to be large because crack self-healing occurred during the experiments. For the results of this study, we define the critical crack width as the “healed crack width” based on the above hypotheses. Figure 10 shows the change in healed crack width owing to self-healing.

Under the concept shown in Figure 10, the residual crack width at healing age t can be expressed as:

(15) $$w_{cr}\left(t\right)=w_{ci}-w_{ch}\left(t\right),$$

where $w_{cr}\left(t\right)$ is the residual crack width at healing age t, $w_{ci}$ is the initial crack width, and $w_{ch}\left(t\right)$ is the healed crack width at healing age t (all in mm). Thus, $w_{ch}\left(0\right)$ is the healed crack width at day 0 of healing age and corresponds to the critical crack width in the literature. Based on this, Equation (10) can be re-written as follows:

(16) $$D_{meas}=\frac{A_{cr}}{A_{tot}}D_{cr}=\frac{w_{cr}\left(t\right)\;d}{A_{tot}}\beta_{cr}\left(t\right)D_{0}=\frac{\left[w_{ci}-w_{ch}\left(t\right)\right]d}{A_{tot}}\beta_{cr}\left(t\right)D_{0}.$$

The coated specimen data in Figure 8 show that, as the healing age increases, the healed crack width increases but the slope of $D_{meas}-D_{ucr}$ as a function of crack width slowly decreases. Because $D_{0}$ is a constant and varies only with the temperature, this decrease in slope can be attributed to the reduction in $\beta_{cr}$. This reduction in crack formation factor appears to originate in the changes in internal crack geometry arising from the irregular formation of self-healing materials in the cracks with increasing healing age and the resulting changes in tortuosity.

#### 4.4.2. Healed Crack Width and Crack Formation Factor

**Figure 11:**
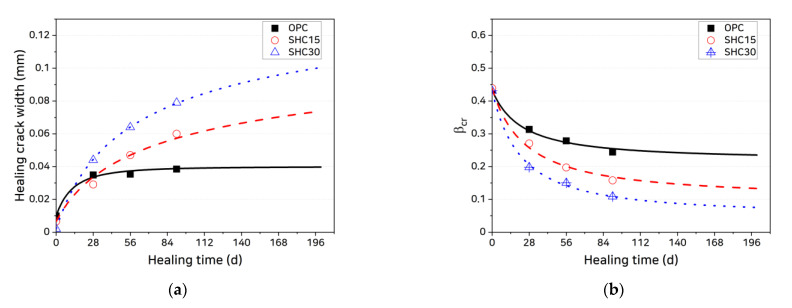
Growth of healed crack width and decrease in $\beta_{cr}$ with increasing healing age: (**a**) healed crack widths and (**b**) $\beta_{cr}.$

The coated specimen test results in Figure 8 can be used to obtain the healed crack width and crack formation factor according to healing age, as shown in Figure 11, from which it is seen that the mixtures containing self-healing materials exhibit larger healed crack widths and lower crack formation factors.

The relationship between healing age and healed crack width and crack formation factor shown in the figure can be expressed as the following hyperbolic functions:

(17) $$w_{ch}\left(t\right)=w_{cc}+\left(w_{chu}-w_{cc}\right)\left[1-{\left(\frac{t_{0}}{t+t_{0}}\right)}^{m}\right]$$

(18) $$\beta_{cr}\left(t\right)=\beta_{crl}+\left(\beta_{cr0}-\beta_{crl}\right){\left(\frac{t_{0}}{t+t_{0}}\right)}^{n},$$

where $t_{0}$ is the age at which healing begins, $w_{chu}$ is the upper limit of the healed crack width, and $\beta_{cr0}$ and $\beta_{crl}$ are the initial value and lower limit of the crack formation factor, respectively. As shown in Figure 11, the two functions closely follow the data trend when the upper limit of the healed crack width ($w_{chu}$) and the lower limit of the crack formation factor ($\beta_{crl}$) are assumed as shown in Table 3. Table 3 lists the main parameters of the healed crack width growth and crack formation factor reduction functions obtained through data fitting.

The healed crack width $w_{ch}\left(t\right)$ and crack formation factor $\beta_{cr}\left(t\right)$ can be defined as intrinsic material properties that depend on the self-healing capacity of the cement-based composite. Using the material parameter values in Table 3 obtained by applying the test method suggested in this study, it is possible to predict the reduction in crack width and the change in the penetration rate of chloride ions through actual cracks as functions of healing age. In test specimens, the crack width hardly changes along the depth, whereas cracks forming in real concrete structures tend to narrow with increasing depth from the surface, making it difficult to evaluate the quantity of chloride ions penetrating through such cracks using test data alone. By applying the healed crack width growth and crack formation factor reduction functions suggested in this study, it is possible to estimate the residual crack width recovered through self-healing. Our results suggest that these models would be useful in evaluating the self-healing-induced recovery of the chloride penetration resistance of cracked concrete in actual structures.

## 5. Conclusions

This study proposed a method of applying coating on uncracked surfaces of test specimens in the electrical migration-diffusion test to improve the evaluation of the recovery of chloride penetration resistance from the self-healing of cracks in cement-based composites. The proposed method has been validated from the experimental data. Based on observations of the self-healing-induced recovery of chloride penetration resistance, a phenomenological model for predicting the progress of crack self-healing in cement-based composites was suggested. The following conclusions were obtained from this study:

1. The time required to reach the quasi-steady state decreases when coating is applied to the uncracked surface of a specimen. This indicates that the application of surface coating can reduce testing time, thereby minimizing the error caused by self-healing during the test.
2. The slope of the crack width-diffusion coefficient relation was found to be higher in the surface-coated specimens. This was attributed to differences between the electrical potentials of the crack and uncracked zone arising from differences in their permittivities. If the electrical potential of crack is assumed to be the same as that of uncracked zone without considering this difference in electrical potential between the two, the diffusion coefficient of the uncracked zone will be overestimated whereas that of the crack will be underestimated, with the degree of error increasing with the crack width. This will in turn result in an overestimation of the self-healing capacity. Thus, the diffusion coefficient of cracked concrete can be evaluated more accurately by applying surface coating.
3. The critical crack width at day 0 of healing age was found to be very small (less than 10 μm) but increased with the healing age. Based on this, it was possible to establish the hypotheses that (a) the critical crack width results from self-healing and (b) the critical crack width is not fixed but varies depending on the healing age, with a rate of change that depends on the self-healing capacity. Based on these hypotheses, the critical crack width can be defined as the “healed crack width” and the diffusion coefficient of a crack can be formulated as a function of the healed crack width and crack formation factor.
4. As self-healing progresses, the healed crack width slowly increases as a result of the formation of self-healing materials in the crack; at the same time, the crack formation factor decreases as the internal crack geometry changes owing to self-healing. The rates of increase in the healed crack width and decrease in the crack formation factor vary depending on the self-healing capacity of the material and can be expressed as hyperbolic functions with upper and lower limits, respectively.
5. By applying the healed crack width growth and crack formation factor reduction functions suggested in this study, it is possible to estimate the residual crack width recovered through self-healing in an actual structure and to predict the chloride ion penetration rate through its cracks. As such, the proposed models are expected to be useful in evaluating the self-healing-induced recovery of the chloride penetration resistance of cracked concrete in actual structures.

## Figures and Tables

**Table 1:** Mixture proportions.

Mixture ID	Proportions							HRWRA (% by Binder Weight)
	Water	OPC	GGBFS	Na_2_SO_4_	Anhydrate Gypsum	Clinker	Fine Aggregate	
OPC	0.4	1	0	0	0	0	2.0	0.5
SHC15	0.4	0.67	0.25	0.015	0.015	0.15	1.90	0.7
SHC30	0.4	0.62	0.25	0.015	0.015	0.30	1.80	0.7

HRWRA = high-range water-reducing admixture.

**Table 2:** Measured crack widths.

Mixture ID	Measured Crack Widths (μm) of Specimens—Mean (Standard Deviations)					
	$w_{cr,target}=100\;\mu m$		$w_{cr,target}=200\;\mu m$		$w_{cr,target}=300\;\mu m$	
	Uncoated	Coated	Uncoated	Coated	Uncoated	Coated
OPC	109.7 (5.91)	101.2 (10.24)	199.6 (9.03)	206.5 (17.90)	298.1 (18.37)	295.3 (12.80)
SHC15	109.9 (7.81)	102.1 (8.02)	202.2 (9.04)	206.9 (11.29)	296.0 (8.70)	299.7 (12.63)
SHC30	97.4 (6.51)	106.9 (7.99)	203.8 (7.44)	203.8 (14.60)	299.2 (10.12)	304.3 (17.55)

$w_{cr,target}$ = target crack width.

**Table 3:** Parameters for healed crack width and crack formation factors.

Mixture ID	Parameters for Healed Crack Width			Parameters for Crack Formation Factor		
	$w_{cc}$	$w_{chu}$	m $\left(R^{2}\right)$	$\beta_{cr0}$	$\beta_{crl}$	n $\left(R^{2}\right)$
OPC	0.009	0.04	2.217 (0.99)	0.433	0.22	1.25 (0.99)
SHC15	0.006	0.15	0.302 (0.99)	0.438	0.10	1.10 (0.99)
SHC30	0.002	0.17	0.420 (0.99)	0.431	0.05	1.29 (0.99)

## Data Availability

Data are contained within the article.
